# Cystic Duct Carcinoma: A New Classification System and the Clinicopathological Features of 62 Patients

**DOI:** 10.3389/fonc.2021.696714

**Published:** 2021-06-11

**Authors:** Lingxi Nan, Changcheng Wang, Yajie Dai, Jie Wang, Xiaobo Bo, Shulong Zhang, Dexiang Zhang, Houbao Liu, Yueqi Wang

**Affiliations:** ^1^ Department of General Surgery, Zhongshan Hospital, Fudan University, Shanghai, China; ^2^ Biliary Tract Diseases Institute, Fudan University, Shanghai, China; ^3^ Cancer Center, Zhongshan Hospital, Fudan University, Shanghai, China; ^4^ Shanghai Key Laboratory of Medical Imaging Computing and Computer Assisted Intervention, Fudan University, Shanghai, China; ^5^ Shanghai Institute of Medical Imaging, Shanghai, China; ^6^ Department of Medical Imaging, Shanghai Medical College, Fudan University, Shanghai, China; ^7^ Department of General Surgery, Xuhui District Central Hospital of Shanghai, Shanghai, China

**Keywords:** cystic duct carcinoma, classification, resectability, prognosis, K-means clustering, t-distributed stochastic neighbor embedding

## Abstract

**Background:**

Cystic duct carcinoma (CDC) is a rare biliary malignancy with a low incidence and poor prognosis. However, the clinical landscape of the disease has not been clarified and no widely applicable classification system has been developed.

**Methods:**

Sixty-two patients with CDC were included in this retrospective study, and a new classification system was established using imaging data. Blood indices, radiological characteristics, pathological features, surgical procedures, and overall survival data were collected. The efficacy of the new classification in predicting resectability was evaluated using receiver operating characteristic (ROC) curves, and K-means clustering and t-distributed stochastic neighbor embedding were applied to verify the conclusion.

**Results:**

The pT stage of patients with type II CDC was significantly worse than that of type I. Patients with type II CDC were more likely to experience distant metastasis and invasion of the nervous system, vascular system, and liver. The resectability of patients with type II CDC was significantly worse than that of patients with type I CDC. Patients with type II CDC had worse prognoses. ROC curve analysis and K-means clustering revealed that the new classification could better categorize patients with CDC than currently available systems.

**Conclusion:**

Patients with type II CDC have significantly worse clinicopathological outcomes. The new classification system has better accuracy in grouping patients with CDC.

## Introduction

Cystic duct carcinoma (CDC) is an extremely rare malignancy in the biliary tract. In 1951, Farrar (1) proposed the following criteria for this rare disease: growth must be restricted to the cystic duct; there can be no neoplastic process in the gallbladder, hepatic, or common bile ducts; and histological examination of the tumor must confirm the presence of carcinoma cells. According to Farrar’s criteria, CDC accounts for only 0.9% of all biliary tract tumors (2). In 2003, Ozden (3) further modified the definition of CDC as a malignant gallbladder lesion that is centered in the cystic duct. Some clinical studies already proved that CDC is a subtype of gallbladder cancer, which is also consistent with the current definition of CDC in the American Joint Committee on Cancer (AJCC) seventh and eighth editions (4, 5).

Over the last two decades, some researchers have proposed their own classifications of CDC (6). In our clinical work, we found that CDC appears to be commonly associated with hilar involvement, and the extent of tumor invasion in the porta hepatis significantly affects resectability and patient prognosis. However, current classifications are not currently suitable for CDC involving the porta hepatis. Among the current classifications, only Yokoyama’s classification defines CDC involving the porta hepatis as the hepatic hilum (HH) type, which still lacks a subdivision based on the extent of invasion (7). We believe that further subdivision of CDC that invades the porta hepatis is necessary to improve the current classification systems.

CDC is a malignant tumor with a poor prognosis (8), and little progress has been made in treatment, especially in the adjuvant setting (9–11). In addition, most studies on primary or metastatic CDC are case reports (12–15) or series with small sample sizes (16–18), and a panoramic view of the clinicopathological features of the disease has not been established. In this study, we clarified the imaging characteristics, clinicopathological features, surgical procedures, and prognosis of CDC by reviewing one of the largest cohorts to date and improved the current classification systems to provide a comprehensive landscape of this rare disease.

## Materials and Methods

### Patients

This retrospective study included 62 consecutive patients with CDC admitted to Zhongshan Hospital (Shanghai, China) between January 2008 and March 2020. All patients were diagnosed *via* preoperative imaging examination, intraoperative exploration, and postoperative pathological reports. Patients in whom the primary site of the tumor could not be confirmed and those whose disease could not be classified according to imaging data were excluded. Patients who had undergone biliary surgery were additionally excluded. This study was approved by the ethics committee of Zhongshan Hospital, Fudan University (Approval No.: B2018-159R).

### Data Extraction

We collected preoperative data for total bilirubin (TBil), direct bilirubin (DBil), alanine aminotransferase (ALT), and aspartate aminotransferase (AST) levels. Preoperative blood indices were based on the results of the first laboratory examination after admission. In particular, for patients undergoing percutaneous transhepatic cholangial drainage (PTCD), preoperative blood indices were measured before PTCD. Preoperative magnetic resonance imaging and computed tomography data were collected. The radiological diagnosis was made by an experienced physician in the Imaging Department of Zhongshan Hospital and reviewed by another physician. The postoperative pathological reports of patients were collected, including the pathological classification of the tumor, lymph node involvement, nervous system invasion, vascular system invasion, and hepatic infiltration. The postoperative pathological results were recorded by two experienced physicians in the Department of Pathology, Zhongshan Hospital. The clinical staging of CDC was performed according to the eighth edition of the AJCC staging manual. We collected the records of surgical procedures, all of which were performed by the chief of the Department of Biliary Surgery at Zhongshan Hospital. The patients were followed-up every 3–6 months for 3 years, and overall survival (OS) data were collected.

### New Classification of CDC

**Figure 1:**
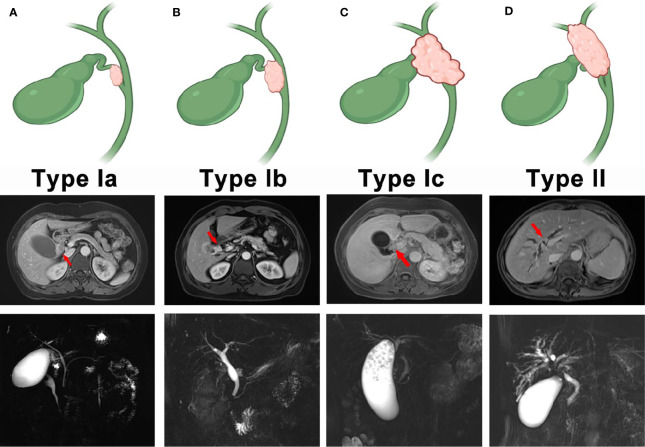
The new classification of cystic duct carcinoma (CDC). **(A)** Type I CDC. The tumor is confined to the cystic duct. **(B)** Type II CDC. The tumor involves part of the extrahepatic bile ducts without causing complete obstruction. **(C)** Type III. The tumor invades the porta hepatis and completely obstructs the common bile duct, but invasion of the confluence of the right and left hepatic ducts does not occur. **(D)** Type IV. The tumor involves the left hepatic duct, right hepatic duct, or the bile ducts above the aforementioned ducts with or without complete obstruction.

In this retrospective study, we established a new classification system that divided CDC into four subtypes. Type Ia CDC refers to a tumor that is confined to the cystic duct without invading other extrahepatic bile ducts (**Figure 1A**). Type Ib CDC describes a tumor involving part of the common bile ducts without causing complete obstruction (**Figure 1B**). Type Ic CDC is a tumor that invades the porta hepatis and causes complete obstruction in the common bile duct without invading the confluence of the right and left hepatic ducts (**Figure 1C**). Type II CDC is defined as a tumor involving the left or right hepatic duct, or the ducts above the aforementioned ducts, with or without complete obstruction of extrahepatic bile ducts (**Figure 1D**). The classification was conducted by an experienced physician in the Imaging Department of Zhongshan Hospital.

### Application of the K-Means Clustering Model and The T-Distributed Stochastic Neighbor Embedding Method

In this study, we clustered all 62 patients using the K-means method (19) based on their surgical characteristics. The results of the clustering were processed using the t-SNE method, which is one of the best methods for dimensionality reduction and visualization of multidimensional models (20). Through dimensionality reduction, we could visualize the distribution of all patients in a two-dimensional plane after clustering. Meanwhile, we compared the level of clustering in the K-means model when the patients were classified by the new classification as well as Yokoyama’s classification, which was assessed using a series of scores including the Calinski–Harabasz score, Davies–Bouldin score, and silhouette score (21). Higher Calinski–Harabasz and silhouette scores and lower Davies–Bouldin scores predicted better clustering.

### Statistical Analysis

SPSS 25 (IBM SPSS Statistics for Windows, Version 25.0, released 2017. IBM Corp., Armonk, NY, USA) and GraphPad Prism 8 (GraphPad Prism 8 for Windows, version 8.0.2. GraphPad Software, San Diego, CA, USA) were used to conduct the analyses. Baseline imaging findings, preoperative blood indices, pathological features, and surgical procedures were compared using Pearson’s chi-squared test, Fisher’s exact test, and one-way analysis of variance. Multiple comparisons were conducted using the least significant difference method. OS curves were drawn using the Kaplan–Meier method, and the log-rank test was used to identify significant differences in survival. When evaluating the two classification systems, receiver operating characteristic (ROC) curves were drawn, and areas under the curve (AUCs) were compared. All statistical tests were two-sided, and P < 0.05 denoted statistical significance.

## Results

### Patient Characteristics

The patients evaluated in this retrospective study were divided into four subtypes according to the new classification system, including 5, 6, 13, and 38 patients with types Ia, Ib, Ic, and II CDC, respectively (**Table 1**). The mean ages of patients with types Ia, Ib, Ic, and II CDC were 64.8, 65.0, 59.5, and 58.6 years, respectively. There was no significant difference in the sex distribution among the subtypes. The pT stage of patients with type II CDC was significantly worse than that of patients with type I CDC (P = 0.012). Furthermore, the pN and pM stages were worse in patients with type II CDC than in those with type I CDC. The TNM stage of patients with type II CDC was significantly worse than that of patients with type I CDC (P = 0.002). Regarding clinical and imaging features, no patients with type Ia or Ib CDC presented with jaundice, whereas most patients with type Ic (P < 0.001) or II CDC (P < 0.001) exhibited this symptom. In terms of imaging features, cholecystectasia and cholecystolithiasis were observed in all four CDC subtypes at similar frequencies. However, cholangiectasis occurred in a minority of patients with type Ia or Ib CDC, but almost all patients with type II CDC presented with cholangiectasis (P < 0.001).

**Table 1:** Baseline of the patients with cystic duct carcinoma.

Variable	New classification				*p*-value	
	Ia	Ib	Ic	II	among type I	type I *vs* II^c^
	(n=5)	(n=6)	(n=13)	(n=38)		
Age (y)				
Mean ± SD	64.8 ± 6.5	65.0 ± 8.9	59.5 ± 9.1	58.6 ± 9.2	0.325^a^	0.162
Gender				
Male	1	2	9	17	0.166^b^	0.686
Female	4	4	4	21		
pT-stage				
T1,2	4	2	4	4	0.204^b^	**0.012**
T3,4	1	4	9	32		
Tx	0	0	0	2		
pN-stage				
N0	3	2	4	8	0.576^b^	0.201
N1,2	2	4	6	19		
Nx	0	0	3	11		
pM-stage				
0	5	5	11	25	0.667^b^	0.057
1	0	1	2	13		
TNM stage				
1	1	1	0	0	0.175^b^	**0.002**
2	2	0	1	0		
3	1	2	7	7		
4	1	3	5	31		
Jaundice				
Present	0	0	10	29	**<0.001** ^b^	**0.006**
Absent	6	6	3	9		
Cholecystectasia				
Present	5	5	10	29	0.780^b^	0.509
Absent	0	1	3	9		
Gallstones				
Present	2	1	11	26	**0.014** ^b^	0.419
Absent	3	5	2	12		
Cholangiectasis				
Present	1	3	10	37	0.098^b^	**<0.001**
Absent	4	3	3	1		

a one way ANOVA (LSD).

b Fisher ‘s exact test (n=24).

c Pearson Chi-Squa (2-sided).

Bold values represent significant p-values (P < 0.05).

### Comparison of Preoperative TBil, DBil, ALT, and AST Levels

**Figure 2:**
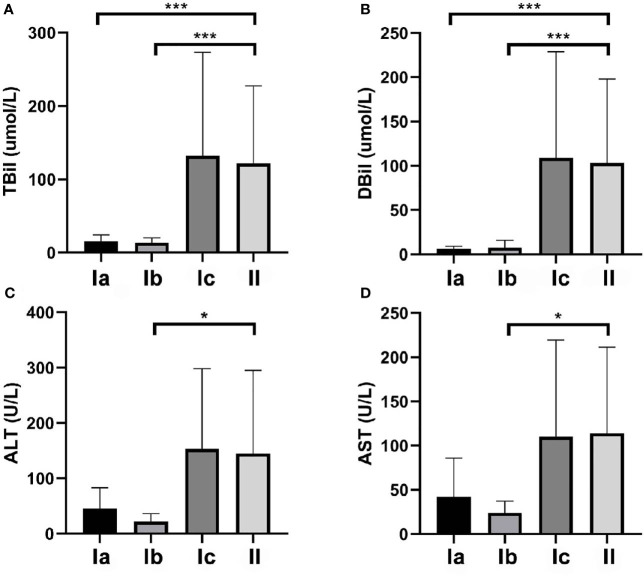
Total bilirubin (TBil), direct bilirubin (DBil), alanine aminotransferase (ALT), and aspartate aminotransferase (AST) levels in the patients. **(A)** The mean preoperative TBil levels in patients with types Ia, Ib, Ic, and II cystic duct carcinoma (CDC) were 15.3, 13.7, 132.1, and 121.7 µmol/L, respectively. The differences between type II and types Ia and Ib were significant (both P < 0.001). **(B)** The mean preoperative DBil levels in patients with types Ia, Ib, Ic, and II CDC were 6.2, 7.7, 108.8, and 103.0 µmol/L, respectively. The differences between type II CDC and types Ia and Ib CDC were significant (both P < 0.001). **(C)** The mean preoperative ALT levels in patients with types Ia, Ib, Ic, and II CDC were 45.8, 21.8, 153.1, and 145.2 U/L, respectively. ALT levels did not differ among the four subtypes. **(D)** The mean preoperative AST levels in patients with types Ia, Ib, Ic, and II CDC were 42.0, 23.8, 110.1, and 114.1 U/L, respectively. AST levels did not differ among the four subtypes. *p < 0.05, ***p < 0.01.

As presented in **Figure 2**, TBil and DBil levels were significantly lower in types Ia and Ib CDC than in type II CDC (both P < 0.001), whereas the differences between patients with types Ic and II CDC were not significant (**Table 2**). However, ALT and AST levels did not differ between types I and II CDC, excluding patients with type Ib CDC (P = 0.046 and P = 0.031, respectively).

**Table 2:** Preoperative indices of the patients with cystic duct carcinoma.

Variable	New classification				*p*-value^a^		
	Ia	Ib	Ic	II	type Ia *vs* Ib	type Ic *vs* II	type Ia+Ib *vs* Ic+II
	(n=5)	(n=6)	(n=13)	(n=38)			
TBil					0.307	0.517	**<0.001**
Mean ± SD	15.34 ± 8.92	13.72 ± 6.45	132.09 ± 141.36	121.73 ± 106.02			
DBil					0.261	0.655	**<0.001**
Mean ± SD	6.16 ± 3.12	7.68 ± 7.98	108.79 ± 119.99	103.01 ± 95.09			
ALT					**0.003**	0.911	**0.021**
Mean ± SD	45.85 ± 37.46	21.75 ± 14.48	153.13 ± 145.40	145.18 ± 150.37			
AST					0.087	0.973	**0.01**
Mean ± SD	42.04 ± 43.84	23.75 ± 13.41	110.11 ± 109.44	114.09 ± 97.26			

a one way analysis of variance (LSD). Bold values represent significant p-values (P < 0.05).

### Pathological Features of Patients With CDC

Regarding pathological features (**Table 3**), most patients with CDC had adenocarcinoma, whereas the remaining patients had other cancer types. The proportion of patients with lymph node involvement was higher for type II CDC (27/38) than for type I CDC (14/24), but the difference was not significant (P = 0.205). Concerning neural invasion, its incidence significantly differed between type Ia CDC (only one patient had neural invasion) and the remaining subtypes, in which most patients had neural invasion. Only one patient each with types Ia and Ic CDC had vascular involvement, versus more than half of patients with type II CDC (18/38), and neural invasion was significant more frequent in type II CDC than in type I CDC (P = 0.040). Hepatic infiltration and distant metastasis only occurred in a small number of patients with type I CDC, but the proportions of patients with liver invasion (18/38) and distant metastasis (15/38) were significantly increased in patients with type II CDC (P = 0.018 and P = 0.041, respectively).

**Table 3:** Pathological features of patients with cystic duct carcinoma.

Variable	New classification				*p*-value	
	Ia	Ib	Ic	II	among type I^a^	type I *vs* II^b^
	n=(5)	n=(6)	n=(13)	n=(38)		
Pathological type				
Adenocarcinoma	4	5	13	37	0.199	0.308
Others	1	1	0	1		
Lymph node involvement				
Present	3	4	7	27	1.000	0.205
Absent	2	2	5	7		
Unknown	0	0	1	4		
Invasion of nervous system				
Present	1	5	11	18	**0.003**	**0.008**
Absent	4	1	0	3		
Unknown	0	0	2	17		
Invasion of vascular system				
Present	1	3	1	18	0.193	**0.040**
Absent	4	3	11	16		
Unknown	0	0	1	4		
Hepatic infiltration				
Present	0	1	2	18	1.000	**0.018**
Absent	5	5	10	19		
Unknown	0	0	1	1		
Distant metastasis				
Present	0	1	2	15	1.000	**0.041**
Absent	5	5	10	23		
Unknown	0	0	1	0		

a Fisher’s exact test (n=24).

b Pearson chi-squared (2-sided). Bold values represent significant p-values (P < 0.05).

### Comparison of Surgical Procedures Between Patients With Types I and II CDC

We collected and analyzed the surgical data of the patients (**Table 4**). All patients with type Ia or Ib CDC underwent radical surgery, as did most of those with type Ic CDC, whereas approximately one-third of patients with type II CDC underwent radical surgery. Compared to the findings in patients with type I CDC, the proportion of patients who underwent radical surgery was much lower in those with type II CDC (P < 0.001). Regarding the surgical margin, there was no significant difference in the proportion of R0 resections between patients with types I and II CDC following radical surgery. The surgical procedures used in patients who underwent radical surgery were similar. All patients underwent cholecystectomy. All but one patient with type Ia CDC underwent extrahepatic bile duct resection, and no patients underwent pancreaticoduodenectomy. The proportion of patients who received hepatectomy did not differ between types I and II CDC.

**Table 4:** Surgical procedures of patients with cystic duct carcinoma.

Variable		New classification				*p*-value	
		Ia	Ib	Ic	II	among type I	type I vs II
		(n=5)	(n=6)	(n=13)	(n=38)		
Operation					
Palliative ^a^		0	0	4	25	0.135^b^	**<0.001** ^c^
Radical		5	6	9	13		
**During radical surgery**		(n=5)	(n=6)	(n=9)	(n=13)		
	Surgical margin				
	R0	4	3	7	9	0.471^b^	1.000^b^
	R1	1	3	2	4		
	Cholecystectomy				
	+	5	6	9	13	/	/
	–	0	0	0	0		
	Extrahepatic bile duct resection				
	+	4	6	9	13	0.250^b^	1.000^b^
	–	1	0	0	0		
	Pancreaticoduodenectomy				
	+	0	0	0	0	/	/
	–	5	6	9	13		
	Hepatectomy ^d^				
	+	3	5	7	9	0.663^b^	1.000^b^
	–	2	1	2	4

a Palliative surgery is performed only with exploratory laparotomy, lymph node biopsy, or palliative cholecystectomy.

b Fisher’s exact test.

c Pearson chi-squared (2-sided).

d All hepatectomy was performed on segment 4b. Bold values represent significant p-values (P < 0.05).

### Comparison of OS Between Patients With Types I and II CDC

**Figure 3:**
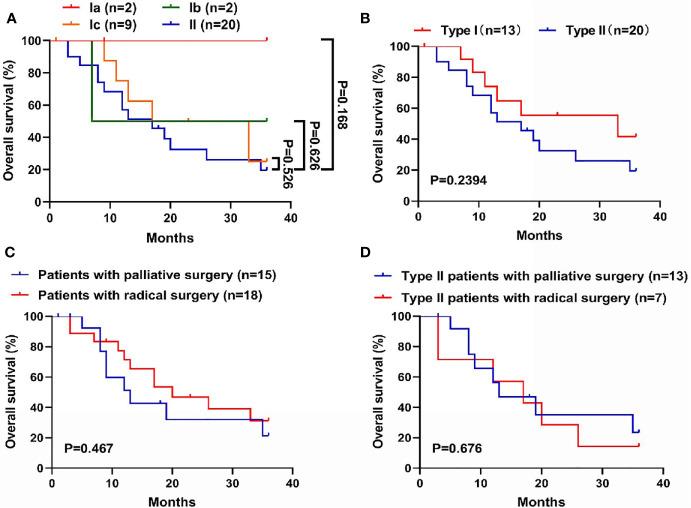
Comparison of overall survival (OS) among the subtypes in the new classification. **(A)** Survival curves of the four subtypes of cystic duct carcinoma (CDC). Prognosis was worst for type II CDC and best for type Ia CDC. **(B)** Survival curves of types I and II CDC. Patients with type I CDC exhibited longer OS (median OS: 33.0 *vs*. 17.0 months), although the difference was not significant. **(C)** Survival curves of CDC patients with radical surgery or palliative surgery. Patients who underwent radical surgery shared a better prognosis, albeit without significance. **(D)** Survival curves of patients with type II CDC, grouped by undergoing radical or palliative surgery. In patients with type II CDC, the benefit of undergoing radical surgery was significantly lower. The mean OS of patients with radical surgery and palliative surgery was 16.7 months *vs*. 19.7 months, respectively.

In total, 33 patients completed follow-up, including 2, 2, 9, and 20 patients with types Ia, Ib, Ic, and II CDC, respectively (**Figure 3A**). Prognosis was best for type Ia CDC and worst for type II CDC. The OS curves differed between types Ic and II CDC, albeit without significance. In general, the median OS was 33.0 months for type I CDC, versus 17.0 months for type II CDC. However, the difference in prognosis between types I and II CDC was not significant (**Figure 3B**).

### Comparison of OS Between Patients With or Without Radical Surgeries

Of the 33 patients who completed follow-up, 18 patients underwent radical surgery, and 15 patients underwent palliative surgery. We compared the prognosis of these patients. In general, the median OS was 20.0 months for patients who received radical surgery, versus 13 months for patients received palliative surgery (**Figure 3C**). However, there was a discrepancy when we merely focused on the patients with type II CDC. Notably, the mean OS came to 16.7 months (95% CI: 8.5-25.0) for patients who underwent radical surgery, versus 19.7 months (95% CI: 12.3-27.1) for patients received palliative surgery (**Figure 3D**).

### Comparison of the New Classification With Yokoyama’s Classification System

**Figure 4:**
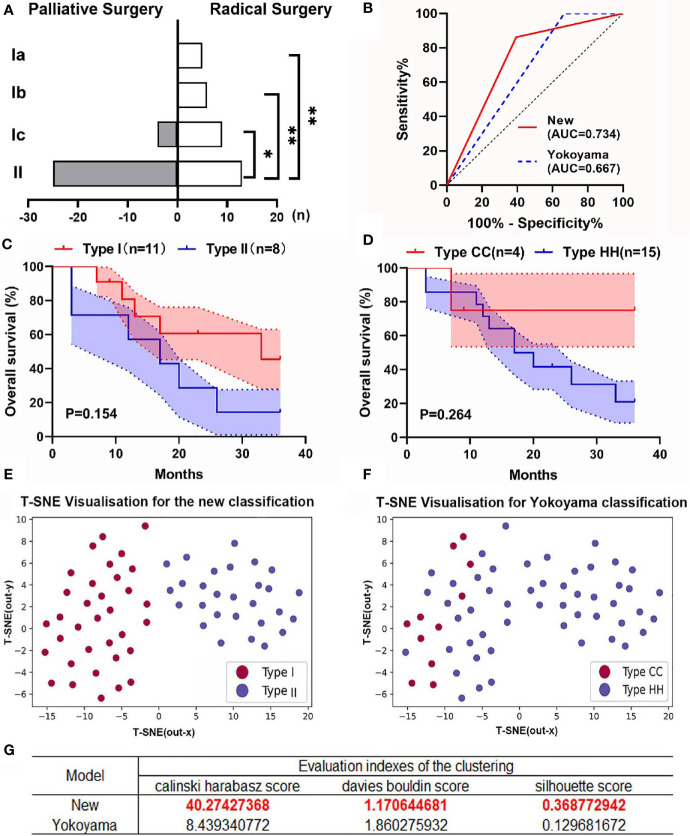
Comparison between the new classification and Yokoyama’s classification. **(A)** Resectability was significantly worse for type II CDC than for type I CDC, whereas the differences among patients with types Ia, Ib, and Ic were not significant. **(B)** The receiver operating characteristic curves of the two classifications for predicting the resectability of patients with CDC. The new classification system had a significantly larger area under the curve (AUC) than Yokoyama’s classification. **(C)** The overall survival (OS) curves of patients with types I (n = 11) and II CDC (n = 8) who underwent radical surgery. The shadows in the figure represent the standard error (SE). **(D)** The OS curves of patients categorized by cystic confluence (CC, n = 4) and hepatic hilum (HH) type CDC (n = 15). All patients underwent radical surgery. The shadows represent the SE. **(E)** The results of K-means clustering with dimensional reduction and visualization using t-distributed stochastic neighbor embedding (t-SNE) in the new classification system. Each point in the figure represents a patient, and its distribution on the two-dimensional plane represents the result of K-means clustering and dimensionality reduction based on the patient’s clinical characteristics. The red dots in the figure represent patients with type I CDC, and the blue dots represent those with type II CDC. **(F)** The results of K-means clustering and t-SNE visualization in Yokoyama’s classification. The red dots in the figure represent patients with type CC CDC, and the blue dots represent those with type HH CDC. **(G)** The evaluation indices of the clustering in the new classification and Yokoyama’s classification. *p < 0.05, **p < 0.001.

Because our classification system and Yokoyama’s system are the only two that define CDC involving the porta hepatis, we compared their predictive utility. According to the criteria of Yokoyama’s classification, type Ic in our system should be included into type HH in Yokoyama’s system. However, because of the significant differences in clinical features between types Ic and II CDC (**Figure 4A**), we believe that our classification is more accurate than Yokoyama’s system. We first compared the efficacy of the two systems in predicting resectability and performed ROC curve analysis (**Figure 4B**). The AUC of the new classification system was significantly higher than that of the Yokoyama system (0.734 *vs*. 0.667), indicating that our classification system more accurately predicted resectability in patients with CDC.

Concerning prognosis, although the rate of radical surgery did not differ between the classification systems (mainly because of the small sample size), the OS curves were more differentiated in the new classification (**Figures 4C, D**).

To validate the aforementioned finding, we conducted K-means clustering, and the results were reduced in dimension and visualized using the t-SNE method (**Figure 4E**). The results suggested that patients with type I CDC were well distributed in the same cluster in the model and clearly distinguished from those with type II CDC, which suggested that the clinical characteristics of CDC significantly differed between types I and II. We then grouped the patients according to Yokoyama’s classification, and the distributions of patients with type HH and cystic confluence (CC) type CDC intersected to some extent (**Figure 4F**). We applied the Calinski–Harabasz, Davies–Bouldin, and silhouette scores to assess the level of clustering in the two classifications. The results suggested that the new classification provided significantly better differentiation than Yokoyama’s classification (**Figure 4G**). These results revealed that type I CDC was distinct from type II CDC in terms of clinical features. The new classification could better categorize patients with CDC and better predict resectability than Yokoyama’s classification.

## Discussion

CDC is a rare tumor of the biliary system that arises along or in the cystic duct (22). The incidence of CDC is low, accounting for only 2.6%–3.3% of bile duct tumors (23, 24). According to autopsy results, primary CDC comprises 0.03%–0.05% of all carcinomas. Because of the extremely low incidence of CDC, little research has clarified its clinical pathological features.

Despite the low incidence, CDC carries the worst prognosis among all biliary tumors (12, 25). The median OS of patients with CDC is only 23 months, which is much lower than that of hilar cholangiocarcinoma or gallbladder carcinoma (26). Surgery is the only radical treatment for CDC, thus highlighting the importance of predicting resectability and prognosis in patients with CDC.

**Figure 5:**
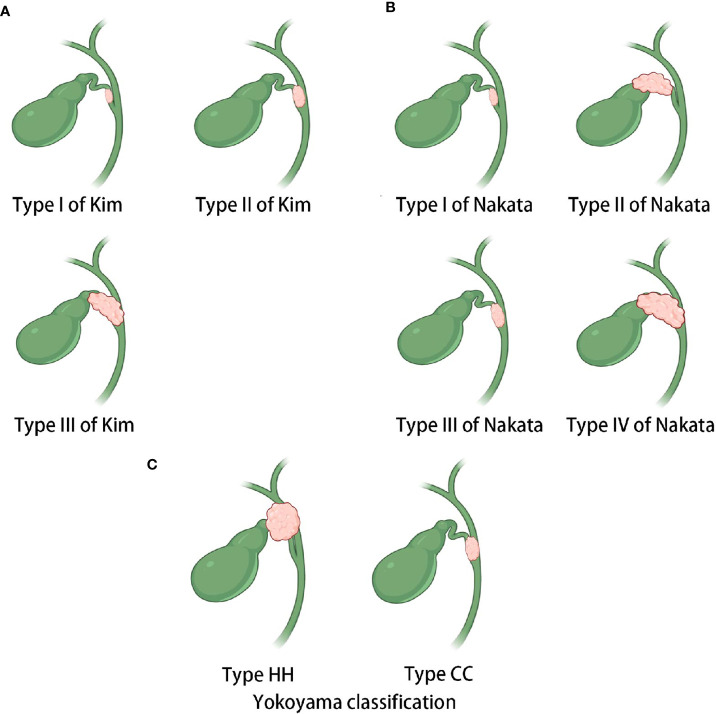
Sketches of the existing classifications of CDC. **(A)** Kim’s classification. Type I carcinoma is restricted to the cystic duct, whereas type II carcinoma involves the neck and infundibulum of the gallbladder or the bile duct of the gallbladder side without obstructive jaundice. Type III carcinoma invades the body of the gallbladder or the contralateral bile duct with obstructive jaundice. **(B)** Nakata’s classification. Type I carcinoma is confined within the cystic duct, whereas type II carcinoma involves the gallbladder. Type III carcinoma involves the common bile duct or common hepatic duct, and type IV carcinoma involves both the bile duct and gallbladder. **(C)** Yokoyama’s classification. The hepatic hilum (HH) type mainly invades the porta hepatis. The cystic confluence (CC) type mainly invades the confluence of the cystic duct.

Currently, the preoperative assessment of resectability and prognosis in patients with CDC is mainly performed using TNM staging and CDC classification systems. However, these approaches have certain drawbacks. On the one hand, there is a lack of a specific TNM staging for CDC, for which staging is mostly based on the criteria for gallbladder cancer in clinical practice. On the other hand, the current CDC classification systems have some limitations. At present, the classifications of Kim (27), Nakata (28), and Yokoyama (7) are widely recognized for CDC (**Figures 5A–C**). However, the classifications of Kim and Nakata do not consider invasion of the porta hepatis in the classification criteria for CDC. Although Yokoyama’s classification includes a separate category for CDC that invades the porta hepatis (type HH), there is no further elaboration according to the extent of tumor involvement in the porta hepatis. In fact, both types Ic and II of the new classification would be categorized as type HH based on Yokoyama’s criteria. However, our findings revealed differences in clinical characteristics between types Ic and II CDC, thus highlighting the need for further subclassification.

In the first part of this study, we provided an overview of the clinicopathological characteristics of patients with CDC by reviewing one of the largest CDC cohort to date. We found that the pT and TNM stages of patients with type II CDC were significantly worse than those of patients with type I CDC. Furthermore, the pathological features of type II CDC significantly differed from those of type I CDC. Invasion of the nervous system, vascular system, and liver together with distant metastasis were significantly more common in type II CDC. More clinically relevant is the fact that the resectability of type II CDC was significantly worse than that of type I CDC. Notably, there was a slight, but not significant, difference in prognosis between types I and II CDC, which could be attributable to the insufficient sample size. Besides, we found that in general, patients with CDC can benefit from radical surgery. However, specifically for patients with type II CDC, radical surgery did not effectively prolong OS. These findings suggest that radical surgery should be carefully considered for patients with type II CDC.

The second part in this study compared the new classification with Yokoyama’s classification because the latter is the only currently available system that considers CDC invading the porta hepatis. The ROC curves suggested that the new classification was superior to Yokoyama’s classification for predicting resectability. However, the systems did not differ in terms of evaluating prognosis. Further, we performed K-means clustering in all patients according to their clinical characteristics and visualized the results using t-SNE. The results indicated that patients with type I CDC were better grouped in the same cluster and clearly distinguished from those with type II in our classification. Conversely, patients with type HH CDC according to Yokoyama’s classification were intermingled with those with type CC CDC, also suggesting that our system is more accurate.

We believe this study holds some potential utility. First, we described the clinical characteristics of CDC using the largest cohort to date, providing a more comprehensive recognition of the disease. Additionally, the new classification can better predict the resectability and prognosis of CDC, which can be of great clinical value for surgeons. However, we should also recognize the shortcomings in the research on CDC. Because of the rarity of CDC, we lack a solid understanding of the pathogenesis of this disease, the pattern of disease progression, and the standardized procedure of surgical treatment. Clinicians often lack reliable guidelines concerning this disease. Therefore, it is essential to conduct a multicenter study and establish agreement in this field.

This retrospective study had some limitations. First, some patients were lost to follow-up. Of all the 62 patients, only 33 patients received complete follow-up, which could be attributed to two causes: first, most of the patients lost to follow-up were admitted for a quite long period of time ago, and second, most of the lost patients were from other provinces. These two factors led to a far more difficult follow-up. To investigate the impact of loss of follow-up on the results of this study, we compared the baseline of patients who received complete follow-up with those who were lost to follow-up (**Table 5**). The results showed a slight difference between the two, indicating that although lost follow-ups inevitably exist, they have a somewhat limited impact on the study findings.

**Table 5:** Baseline of the patients with or without complete follow-up.

Variables	Patients with complete follow-up		Patients without complete follow-up	*p*-value
	(n=33)		(n=29)	
Age (y)			
Mean ± SD	60.8 ± 9.6		59.0 ± 8.6	0.514^a^	
Gender			
Male	18		11	0.191^b^	
Female	15		18		
Pathological type			
Adenocarcinoma	31		28	0.632^b^	
Others	2		1		
pT-stage			
T1,2	10		4	0.115^b^	
T3,4	23		23		
Tx	0		2		
pN-stage			
N0	11		6	0.204^b^	
N1,2	13		18		
Nx	9		5		
pM-stage			
0	25		21	0.764^b^	
1	8		8		
TNM stage			
1	2		0	0.523^b^	
2	2		1		
3	8		9		
4	21		19		
Lymph node involvement			
Present	10		6	0.616^b^	
Absent	21		20		
Unknown	2		3		
Invasion of nervous system			
Present	2		6	0.230^b^	
Absent	20		15		
Unknown	11		8		
Invasion of vascular system			
Present	23		11	**0.032** ^b^	
Absent	9		14		
Unknown	1		4		
Hepatic infiltration			
Present	22		17	0.295^b^	
Absent	11		10		
Unknown	0		2		
Distant metastasis			
Present	24		19	0.515^b^	
Absent	9		9		
Unknown	0		1		
Operation			
Palliative	15		14	0.824^b^	
Radical	18		15		
Jaundice			
Present	13		10	0.690^b^
Absent	20		19
CDC type			
Type I	13		11	0.906^b^	
Type II	20		18

a Student’s t test.

b Pearson Chi-Squa (2-sided). Bold values represent significant p-values (P < 0.05).

Besides, although this study featured one of the largest cohorts of patients with CDC to date, the overall sample size was rather small (especially for types Ia and Ib), which increased the statistical bias. Although the prognosis of type II CDC was clearly worse than that of type I CDC, it is not possible to clarify the survival differences among the type I subtypes, especially type Ic in comparison to types Ia and Ib. These differences could be meaningful, and further investigation is required. Currently, we are enrolling patients with CDC to expand the sample size, and we hope to provide a more comprehensive description of this rare tumor.

## Conclusions

In this retrospective study, 62 patients with CDC were consecutively included. We assessed blood indices, radiology characteristics, pathological features, surgical procedures, and prognoses for these 62 patients to present an overview of this rare disease using one of the largest CDC cohorts to date. Meanwhile, we established a new classification system to overcome the shortcomings of the current systems. We found that the new classification could better categorize patients with CDC than currently available systems, and this finding was validated by the results of K-means clustering and t-SNE.

## Data Availability Statement

The original contributions presented in the study are included in the article/supplementary material. Further inquiries can be directed to the corresponding authors.

## Ethics Statement

Written informed consent was obtained from the individual(s) for the publication of any potentially identifiable images or data included in this article.

## Author Contributions

All authors contributed to the article and approved the submitted version. LN, CW, and YD for acquisition of data, analysis and interpretation of data, statistical analysis, and drafting of the manuscript. JW, XB, SZ, and DZ for technical and material support. YW and HL for study concept and design, analysis and interpretation of data, drafting of the manuscript, obtained funding, and study supervision.

## Funding

This study was supported by grants from the National Natural Science Foundation of China (81872352, 82002525), the Foundation of Shanghai Science and Technology Committee (20JC1418902), the Shanghai Sailing Program (20YF1407300), the JianFeng project of XuHui Provincial Commission of Health and Family Planning (SHXH201703), the Clinical Study of Zhongshan Hospital (2018ZSLC24), the scientific research project of Shanghai Municipal Health Commission (201940389), the Youth Foundation of Zhongshan Hospital (2020ZSQN61), and the Resident Foundation of Zhongshan Hospital (ZPJJ-092).

## Conflict of Interest

The authors declare that the research was conducted in the absence of any commercial or financial relationships that could be construed as a potential conflict of interest.
